# Neurofilament light as a biomarker for motor decline in Parkinson’s disease

**DOI:** 10.3389/fnins.2022.959261

**Published:** 2022-09-01

**Authors:** Yumei Liu, Kaixin Dou, Ling Xue, Xiaoyuan Li, Anmu Xie

**Affiliations:** ^1^Department of Neurology, Affiliated Hospital of Qingdao University, Qingdao, China; ^2^Department of Nursing, Tai’an City Central Hospital, Tai’an, China; ^3^Department of Traditional Chinese Medicine, Affiliated Hospital of Qingdao University, Qingdao, China

**Keywords:** Parkinson’s disease, neurofilament light, motor impairment, α-syn, regression analysis, mediating effect analysis

## Abstract

**Objectives:**

The aim of this study was to determine whether neurofifilament light (NfL) could reflect motor decline and compare the predictive values of cerebrospinal fluid (CSF) and serum NfL in individuals with PD.

**Methods:**

CSF/serum samples were collected from patients with PD and healthy controls (HCs) with motor assessments at baseline and after three years of follow-up from the Parkinson’s Progression Markers Initiative (PPMI). Multiple linear regression models and linear mixed-effects models were used to investigate the associations of motor assessments with baseline and longitudinal CSF/serum NfL. Associations between the change rates of motor assessments and CSF/serum NfL were further investigated *via* multiple linear regression models. Mediating effect analysis was used to research whether CSF alpha-synuclein (α-syn) acts as the mediator between NfL and motor assessments.

**Results:**

We found patients with PD had higher baseline CSF/serum NfL levels than HCs. Both baseline CSF/serum NfLs and their change rates predicted measurable motor decline in PD assessed by different motor scores. Baseline serum NfL and its rate of change were strongly associated with CSF NfL levels in patients with PD (*P* < 0.001). Besides, there were also significant differences in CSF/serum NfL levels and predicted values of motor decline between men and women with PD. Mediating effect analysis showed CSF α-syn mediated the effect of CSF NfL on total Unified Parkinson’s Disease Rating Scale (UPDRS) scores and UPDRSIII with 30.6 and 20.2% mediation, respectively.

**Conclusion:**

Our results indicated that NfL, especially serum NfL concentration, could serve as an easily accessible biomarker to monitor the severity and progression of motor decline in individuals with PD, especially in men with PD. Besides, CSF α-syn acts as a mediator between NfL and motor progression.

## Introduction

Parkinson’s disease (PD) is a complex progressive neurodegenerative disease that affects more than 1% of the worldwide population. Pathologically, PD is characterized by loss of dopaminergic neurons in the substantia nigra pars compacta (SN) and abnormal aggregation of Lewy bodies. Although current treatment offers symptomatic benefits for motor symptoms of PD such as resting tremor, cogwheel rigidity, and bradykinesia, they do not prevent disease progression. Besides, the expected mortality of PD is two- to threefold higher compared with the general population. Therefore, it is essential to find an easily accessible biomarker that could reflect the process of neuronal degeneration to predict disease progression and possibly as an objective measure for future mechanism-targeted therapeutic responses.

In recent decades, several biomarkers have been found to be diagnostic of PD. An increase in cerebrospinal fluid (CSF) alpha-synuclein (α-syn) was association with further deterioration of motor and cognitive deficits (Hall et al., 2015; Majbour et al., 2016). CSF soluble fragment of triggering receptor expressed on myeloid cells 2 (sTREM2) may be a promising predictor for the cognitive decline in PD rather than a diagnostic biomarker (Qin et al., 2022). CSF GCase activity has been shown to be significantly reduced in PD compared to controls (Paciotti et al., 2019; Parnetti et al., 2019). The p-tau/α-syn ratio combined with TNF-α could separate patients with PD from controls (Balducci et al., 2007).

Neurofifilament light (NfL) is a neuron-specific protein component involved in the assembly and maintenance of the axonal cytoskeleton, which is elevated in CSF and serum due to axonal damage and neurodegeneration (Khalil et al., 2018). Growing evidence indicates that blood NfL levels reflect disease progression in patients with PD (Mollenhauer et al., 2020; Ye et al., 2021). Besides, higher serum and CSF NfL levels in patients with PD have been shown to correlate with longer disease duration, greater disease severity, and a higher risk of motor symptom progression (Lin et al., 2019; Mollenhauer et al., 2020; Oosterveld et al., 2020). However, NfL has not been evaluated as a predictor of motor decline, although previous reports have suggested that this marker is associated with motor decline (Ye et al., 2021). Therefore, we aimed to explore whether increases in NfL levels could reflect motor decline in patients with PD from the Parkinson’s Progression Markers Initiative (PPMI) and compare the predictive value of NfL in serum and CSF. First, we studied the relationship between NfL and motor function scores both cross-sectionally and longitudinally. Then, we performed mediation analysis of other biomarkers of neurodegeneration, aiming to disentangle the pathological mechanisms between NfL and motor decline.

## Materials and methods

### Study design

In short, PPMI is an ongoing observational, international, prospective, longitudinal, and multicenter study to identify serological, genetic, CSF, and imaging biomarkers of PD progression. The purpose and methods of this study have been published elsewhere and are available at www.ppmi-info.org/study-design (Parkinson Progression Marker Initiative, 2011). The data used in this article were an analysis of baseline and 3-year follow-up data downloaded on 4 March 2021. All included subjects who remained in the study had completed at least 3 years of follow-up at the time of data download.

### Participants

Inclusion and exclusion criteria have been published elsewhere (Parkinson Progression Marker Initiative, 2011). Enrollment criteria for *de novo* participants with PD in PPMI were listed as follows: (1) age older than 30 years; (2) diagnosis of PD within 2 years; (3) the presence of two of the following symptoms: bradykinesia, rigidity, and resting tremor; (4) untreated for PD at the baseline visit; (5) be in Hoehn and Yahr stage 1 or 2; and (6) 123-I isoflurane DaT imaging in patients with PD reveals dopamine transporter deficiency. To prevent misdiagnosis, we conducted a longitudinal review of the diagnosis. Patients should be excluded from follow-up if PSP, MSA, or other neurological disorders are suspected. Healthy controls (HCs) were required to have no significant neurological impairment; all first-degree family members without PD were age and gender matched with PD. Participants included in this study needed to meet the following criteria: they had no missing baseline NfL and had at least one additional NfL monitoring during the last 3 years [excluding 4 CSF samples (HC:PD = 0:4) and 11 serum samples (HC:PD = 2:9) without follow-up data].

### Serum and cerebrospinal fluid neurofilament light analysis

Serum and CSF NfL were measured with the Simoa Human Nf-Light Advantage kit using a fully automated SIMOA^®^ HD-1 analyzer based on a Single-Molecule Array technology (Quanterix, Lexington, MA, United States) (Sampedro et al., 2020). Quantitative measurements of NfL in blood by Simoa technology have been shown to be reliable (Soylu-Kucharz et al., 2017). More details on the processing of samples in the cohort can be read in the biologic’s manual for PPMI^1^ (Kang et al., 2013).

### Cerebrospinal fluid alpha-synuclein analysis

Cerebrospinal fluid was collected using standardized lumbar puncture procedures. Sample collection and processing were carried out according to the PPMI biologics manual (see text footnote 1) and described elsewhere (Kang et al., 2016). Sandwich-type immunoassays (BioLegend, San Diego, CA, United States, formerly Covance) were used to quantify CSF α-Syn. All measurements were performed in singlicate on a cobas e 411 analyzer at Covance Greenfield Laboratories (Translational Biomarker Solutions, IN, United States).

### Clinical assessment measures

Clinical assessment methods are described in detail on the PPMI website and published earlier. From enrollment up to 3 years, PD-related signs and symptoms were assessed annually by the Movement Disorders Society-sponsored Unified Parkinson’s Disease Rating Scale (UPDRS). MDS-UPDRS III inferred motor status. Postural instability and gait difficulty (PIGD) and tremor scores were calculated due to previously defined criteria (Stebbins et al., 2013). The PIGD measure includes five items, which are freezing, walking, and balance in UPDRS part 2 (UPDRS-II), gait and freezing of gait, and postural stability in UPDRS-III. Tremor measure includes 11 items, including tremor in UPDRS-II, postural tremor, kinetic tremor, rest tremor, and rest constancy in UPDRS-III. Akinetic-rigid scores were computed by adding eight items, including rigidity, finger tapping, hand movements, pronation-supination movements of hands, toe tapping, leg agility, arising from the chair, and body bradykinesia in UPDRS-III (Kang et al., 2005). All subjects underwent DatScan to measure the dopamine transporter (DAT) analyzed according to the imaging technical operations manual (see text footnote 1). DaTscan was used to perform dopamine imaging in standardized methods (Parkinson Progression Marker Initiative, 2011). Quantitative DaTscan measures in the striatal binding ratio (SBR) of caudate, putamen, or striatal uptake were used in our analyses.

### Statistical analysis

The CSF or serum NfL levels did not show a normal distribution (Kolmogorov–Smirnov test: *P* < 0.01). Thus, the related data were transformed to obtain a normal distribution using R software and then used in the following analyses. Outliers were defined as three standard deviations (SDs) below or above the mean and were excluded to eliminate the influence of extreme values [excluding five CSF samples (HC:PD = 1:4) and five serum samples (HC:PD = 2:3)]. The groups were compared using Wilcoxon rank-sum tests (for two groups). In the cross-sectional analyses, multiple linear regression models were run separately for combinations of each motor measure and normalized NfL levels. In the longitudinal analyses, the linear mixed-effects (LME) model was used to test for changes over time in CSF and serum NfL levels separately by the group. Besides, we investigated the relationships between change rates of NfL levels/baseline NfL levels and longitudinal changes in the scores of motor measurements *via* multiple linear regression models. The mean change rates (estimated by the sim function in the “arm” package with 10,000 replicates) in both NfL levels and scores of motor items among the whole samples were computed *via* LME models. Mediation analyses were performed with the package “mediation” in R. The linear regression models tested the effects of CSF α-syn on motor features. Baron and Kenny (1986) proposed the methods that supported the linear regression models fitted. All statistical analyses were performed using the R software. We conducted the following steps to identify the adjustment factors. First, the univariate relationship between each predictor and the NfL level was examined. Then, any variables that had univariate associations with a *P*-value < 0.2 were included in a multivariable model. We finally determined gender and tau values as adjustment variables.

## Results

### Baseline characteristics

**FIGURE 1:**
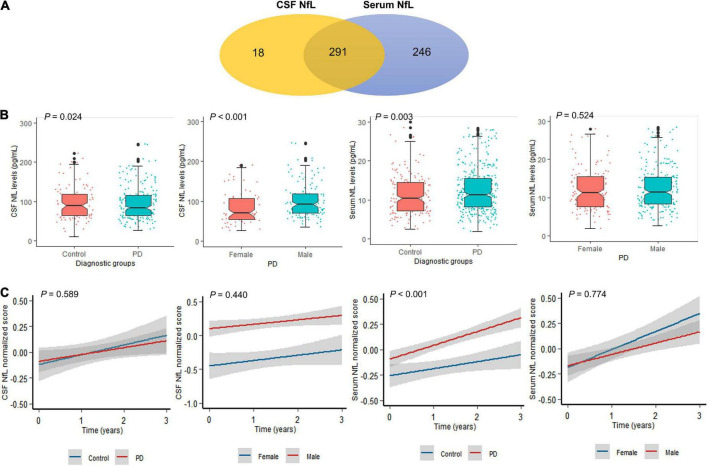
Neurofilament light (NfL) levels between controls and patients with PD. **(A)** A total of 555 participants provided one serum and/or CSF sample for NfL measurement. A total of 291 participants provided both CSF and serum samples. **(B)** Baseline NfL levels of controls and patients with PD. **(C)** Longitudinal NfL values of controls and patients with PD.

Participant’s demographic information and clinical features at baseline are listed in Table 1. A total of 291 individuals provided samples for NfL measurement from both serum and CSF, 246 from serum alone and 18 from CSF alone (Figure 1A). CSF NfL study included 207 individuals with PD and 102 HCs. Serum NfL study enrolled 361 individuals with PD and 176 HCs. There is no difference in age, sex, or education status between PD and HC groups. As we can see, patients with PD had lower α-syn, t-tau, mean caudate SBR values, mean putamen SBR values, and mean striatum SBR values than controls. More importantly, baseline CSF NfL levels were higher in the individuals with PD (99.9 pg/ml) than controls (98.7 pg/ml) (*P* = 0.024) [Figure 1B(a)], and CSF NfL levels were significantly elevated in men with PD (101 pg/ml) compared to women with PD (83.4 pg/ml) (*P* < 0.001) [Figure 1B(b)]. Baseline serum NfL levels were higher in PD groups (12.5 pg/ml) than controls (11.5 pg/ml) (*P* = 0.003) [Figure 1B(c)]. Nevertheless, there is no difference in serum NfL levels between men and women with PD (*P* = 0.524) [Figure 1B(d)].

**TABLE 1:** Baseline demographics and clinical characteristics.

	CSF		*P*	Serum		*P*
	PD	HC		PD	HC	
No. of individuals (*N*)	207	102		361	176	
**Age**						
Mean years (SD)	61 (9.96)	61.7 (10.9)	0.43	61.7 (9.8)	60.8 (11.2)	0.47
(Min, max)	(33, 85)	(31, 83.7)		(33.5, 84.9)	(30.6, 83.7)	
Missing	0	0		0	0	
**Gender (%)**						
Men	135 (65.2%)	66 (64.7%)	0.84	231 (64%)	113 (64.6%)	0.82
Women	72 (34.8%)	36 (35.3%)		130 (32%)	63 (35.4%)	
**Education (y)**						
Mean (SD)	16 (2.6)	16.2 (2.6)	0.49	15.5 (3)	16.1 (2.9)	0.056
(Min, max)	(10, 26)	(11,23)		(5, 26)	(8, 23)	
Missing	0	0		0	0	
**Mean caudate**						
Mean (SD)	2 (0.6)	2.9 (0.6)	<0.001	2 (0.5)	3.0 (0.6)	<0.001
(Min, max)	(0.6, 3.7)	(1.6, 5.2)		(0.4, 3.7)	(1.3, 4.8)	
Missing	2	1		3	1	
**Mean putamen**						
Mean (SD)	0.8 (0.3)	2.1 (0.6)	<0.001	0.8 (0.3)	2.1 (0.5)	<0.001
(Min, max)	(0.3, 1.9)	(0.9, 3.9)		(0.2, 2.2)	(0.6, 3.9)	
Missing	2	1		3	1	
**Mean striatum**						
Mean (SD)	1.4 (0.4)	2.5 (0.6)	<0.001	1.4 (0.4)	2.6 (0.6)	<0.001
(Min, max)	(0.4, 2.6)	(1.2, 4.2)		(0.3, 2.6)	(0.98, 4.24)	
Missing	2	1		3	1	
**α-Syn (pg/ml)**						
Mean (SD)	1,504.4 (711)	1,675.8 (736.4)	0.02	1,524.9,(686.3)	1,688 (733.5)	0.006
(Min, max)	472 (5,256)	600.6 (4,148.4)		432.4 (5,256.9)	488.6 (4,683.1)	
Missing	0	0		7	5	
**Tau (pg/ml)**						
Mean (SD)	167 (55.4)	192.8 (79)	0.007	169.3 (56.8)	193.9 (79.4)	0.002
(Min, max)	(83.93, 345.3)	(81.96, 580.8)		(80.93, 467)	(81.96, 580.8)	
Missing	6	1		15	8	

PD, Parkinson’s disease patients; HC, healthy controls; CSF, cerebrospinal fluid; NfL, neurofilament light.

### Cross-sectional neurofilament light analyses

**FIGURE 2:**
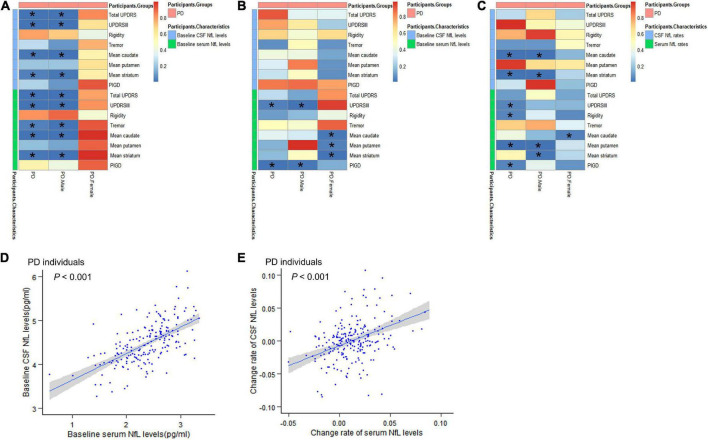
Associations between motor features and NfL levels in cross-sectional and longitudinal analyses. **(A)** Associations between motor features and NfL levels in cross-sectional analyses. **(B)** Effects of baseline NfL levels on biomarkers and motor features among participants with PD during follow-up. **(C)** Associations between longitudinal changes in scores of motor features and change rates of NfL levels. **(D)** The relationship between baseline serum NfL levels and baseline CSF NfL levels. **(E)** The relationship between change rates of serum NfL levels and change rates of CSF NfL levels. CSF, cerebrospinal fluid; PD, Parkinson’s disease patients; NfL, neurofilament light; UPDRS, Unified Parkinson’s Disease Rating Scale; PIGD, postural instability and gait disorder. *P < 0.05.

Multiple linear relationships between motor features and CSF/serum NfL after adjustment for gender and tau are shown in Figure 2A and Supplementary Table 1. Correlation between baseline CSF NfL levels and motor severity, which was assessed by total UPDRS scores (β = 0.223, *P* = 0.008), UPDRSIII (β = 0.218, *P* = 0.009), mean caudate SBR values (β = −0.118, *P* = 0.019), and mean striatum SBR values (β = −0.107, *P* = 0.034), remained significant in PD groups. Interestingly, compared with women with PD, there were significant relationships between baseline CSF NfL levels and total UPDRS scores (β = 0.392, *P* < 0.001), UPDRSIII (β = 0.357, *P* < 0.001), mean caudate SBR values (β = −0.182, *P* = 0.008), and mean striatum SBR values (β = −0.165, *P* = 0.019) in men with PD.

The effect of baseline serum NfL on motor decline (total UPDRS scores, UPDRS-III, mean caudate SBR values, and mean striatum SBR values) in PD groups was similar with CSF NfL. Besides, we also found the association between serum NfL levels and tremor (β = 0.174, *P* = 0.035). And we found that the correlation between serum NfL levels and motor severity assessed by total UPDRS scores (β = 0.219, *P* < 0.001), UPDRSIII (β = 0.228, *P* < 0.001), tremor (β = 0.286, *P* = 0.008), mean caudate SBR values (β = −0.136, *P* = 0.001), and mean striatum SBR values (β = −0.121, *P* = 0.004) remained significant in men with PD.

### Longitudinal neurofilament light values

Cerebrospinal fluid NfL levels did not differ between the PD and control groups across all visits (*P* = 0.589) [Figure 1C(a)]. Serum NfL levels were also higher in PD than control groups across all visits (*P* < 0.001) [Figure 1C(c)]. Longitudinal changes were not significant restricted to men and women with PD in CSF (*P* = 0.440) and serum NfL analyses (*P* = 0.774) [Figure 1C(b,d)].

### Prediction of longitudinal changes of motor decline using baseline neurofilament light levels

We performed an exploratory analysis based on previous studies on biomarkers to analyze the predictive value of biomarkers in PD motor features. Hypothetically, the upregulation of NfL would be associated with a decrease in overall motor performance. In patients with PD, baseline CSF NfL levels were not related to longitudinal changes of any motor indicators (Figure 2B and Supplementary Table 2).

For serum NfL, higher baseline NfL predicted a higher increased rate of UPDRSIII score (β = 0.344, *P* = 0.038) and PIGD score (β = 0.118, *P* = 0.005) in patients with PD. In men with PD, higher baseline serum NfL levels were also associated with greater motor decline, as measured by UPDRSIII (β = 0.532, *P* = 0.013) and PIGD (β = 0.107, *P* = 0.018). Elevated levels of serum NfL at baseline were associated with a significantly faster decrease in mean caudate SBR values (β = −0.033, *P* = 0.021), mean putamen SBR values (β = −0.018, *P* = 0.038), and mean striatum SBR values (β = −0.028, *P* = 0.01) in women with PD (Figure 2B). Most importantly, serum and CSF values of baseline NfL are significantly correlated (β = 0.615, *P* < 0.001) (Figure 2D and Supplementary Table 3).

### Prediction of longitudinal changes of motor decline using longitudinal neurofilament light

Associations between longitudinal changes in scores of motor function and change rates of NfL levels are shown in Figure 2C and Supplementary Table 4. Compared with results shown in baseline NfL analyses, a longitudinal 3-year increase in CSF NfL levels was also significantly associated with motor impairments, which were tested by mean caudate SBR values (β = −0.516, *P* < 0.001) and mean striatum SBR values (β = −0.269, *P* = 0.002) in PD groups. However, there is no meaningful association between change rates of CSF NfL levels and longitudinal changes in motor performance scores in women with PD compared to men with PD.

Change rates of serum NfL levels were related to changes in scores of UPDRS III (β = 7.841, *P* = 0.048), rigidity (β = 2.337, *P* = 0.042), mean putamen SBR values (β = 0.32, *P* = 0.003) and PIGD (β = 1.934, *P* = 0.044), and correlated with change rates of CSF NfL levels (β = 0.640, *P* < 0.001) in PD groups (Figure 2E and Supplementary Table 3). In men with PD, there was a positive association between change rates of serum NfL levels and longitudinal changes in scores of mean putamen SBR values (β = 0.425, *P* = 0.002) and mean striatum SBR values (β = 0.353, *P* = 0.017). However, there was a significant negative correlation between serum NfL rate and mean caudate SBR values rate (β = −0.627, *P* = 0.031) in women with PD.

### Cerebrospinal fluid alpha-synuclein statistically mediates the association between neurofilament light and motor characteristics

**FIGURE 3:**
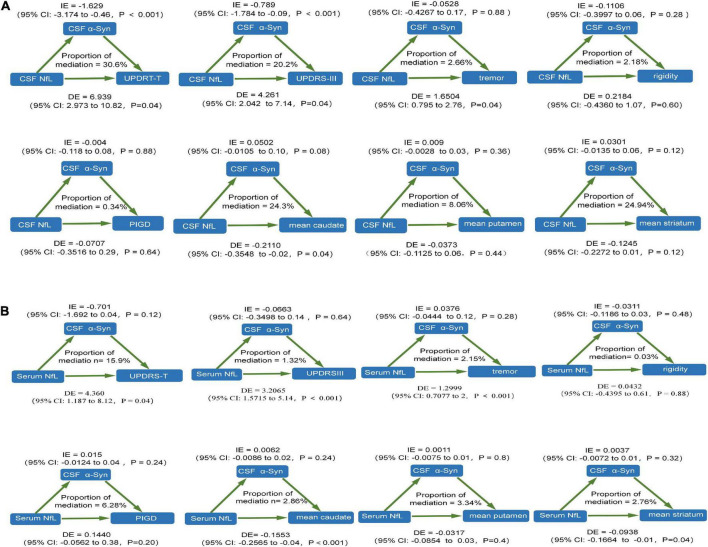
The relationship between NfL levels and motor features was mediated by CSF α-syn. **(A)** CSF NfL levels. **(B)** Serum NfL levels. CSF, cerebrospinal fluid; PD, Parkinson’s disease patients; NfL, neurofilament light; UPDRS, Unified Parkinson’s Disease Rating Scale; PIGD, postural instability and gait disorder.

We performed mediation analyses to investigate whether NfL contributed to the prediction of motor symptoms of PD as assessed by motor characteristics *via* α-syn. We found that CSF α-syn mediated the effect of baseline CSF NfL on total UPDRS scores and UPDRSIII at baseline with 30.6 and 20.2% mediation, respectively. However, there was no mediating effect on tremor, rigidity, PIGD, mean caudate SBR values, mean putamen SBR values, and mean striatum SBR values at baseline (Figure 3A). There was no mediation for the influence of baseline serum NfL on motor characteristics (Figure 3B).

## Discussion

This study demonstrated that NfL levels in the PD group was significantly higher than in the control group, which is consistent with previous cross-sectional studies (Ma et al., 2021). Notably, the study showed that NfL levels could reflect disease severity in terms of motor function in patients with PD from cross-sectional and prospective follow-up research for the first time. Baseline CSF and serum NfL levels were found to predict the decline in overall motor functions, worsening of total UPDRS scores, UPDRSIII scores, and tremor scores but not rigidity at baseline in individuals with PD, which is consistent with previous cross-sectional studies (Backstrom et al., 2020). Indeed, previous longitudinal studies have shown that higher NfL levels in blood and CSF were related to an increased risk of PD motor progression (Lin et al., 2019). In this three-year longitudinal study of PD, we also demonstrated that a higher increasing rate of CSF and serum NfL levels could predict more rapid individual motor decline progression among patients with PD assessed by different indicators. Moreover, we found that an increasing rate of serum NfL could better predict the motor impairments based on our results.

Recent studies have researched the potential relationship between DAT uptake and NfL levels, but results have been inconsistent (Backstrom et al., 2020; Sampedro et al., 2020; Ye et al., 2021). In our study, we found higher baseline NfL levels in CSF and serum both have a negative effect on caudate and striatum DAT binding ratio in patients with PD. Surprisingly, our study found a higher increasing rate of CSF NfL has a negative relationship with caudate and striatum DAT binding ratio in patients with PD, which is consistent with the previous study (Backstrom et al., 2020). But the greater change in serum NfL levels was associated with the more remarkable growth of the putamen DAT binding ratio over time. This may indicate that CSF NfL is a supplement to serum NfL in predicting motor performance and requires further research. The exact mechanism is unclear. Consistently, SPECT and PET studies in PD showed that denervation of dopaminergic neurons was more pronounced in the putamen than in the caudate nucleus. The caudate nucleus is affected later due to disease progression (Brooks and Piccini, 2006; Jakobson Mo et al., 2013). A study has shown that early denervation in the caudate nucleus is associated with higher CSF NfL compared to the putamen and may be a more sensitive marker of rapidly progressive neurodegenerative diseases (Backstrom et al., 2020). Besides, previous cross-sectional and longitudinal results indicated striatal DAT binding ratio was linked to bradykinesia and rigidity in PD (Rossi et al., 2010; Li et al., 2018).

This is the first time that NfL expression and predictive value differ between men and women with PD. Intergroup comparisons in the study indicated that baseline CSF NfL levels were attenuated in women with PD compared with men (*P* < 0.001). However, there were no significant results in serum samples (*P* = 0.774). And our study found NfL, especially serum NfL levels, could better predict motor symptom progression in men with PD. The precise mechanism remains unclear. One explanation is that both the incidence and prevalence of PD are 1.5–2 times higher in men than in women (Benito-Leon et al., 2003). Besides, onset in women was slightly later than in men, and women had better lower UPDRS motor scores but a greater prevalence of dyskinesias compared with men (Haaxma et al., 2007). In our study, UPDRS motor scores of women with PD were lower than those of men with PD at baseline, which is consistent with previous study (Supplementary Table 1; Kang et al., 2022). These may be due to higher dopaminergic activity at baseline and a possible protective influence of estrogens related to different iron levels in women with PD (Jurado-Coronel et al., 2018). Thus, relationships between NfL and motor features could not be shown in the women with PD.

Neurofilament proteins are capital components of the axonal cytoskeleton and are especially abundant in axons composed of four subunits: light chain, medium chain, heavy chain, and α-internexin. When axons are damaged, NfL is released and then pulled into interstitial fluid, drained toward the CSF, and later to peripheral blood (Khalil et al., 2018). Human postmortem brain studies have shown that axonal degeneration is a prominent feature in early-stage PD (Moors et al., 2021). Increasing NfL concentrations in the CSF and serum is an indicator of neuronal/axonal injury and degeneration (Gafson et al., 2020). However, there are still contradictions in the alterations of CSF and blood NfL of patients with PD from existing studies. A study showed that serum NfL is strongly associated with CSF NfL levels in patients with PD (Marques et al., 2019). Besides, the correlation coefficient between cerebrospinal fluid and plasma/serum NfL was as high as 0.86–0.94, suggesting a strong correlation between CSF and plasma NfL, indicating that most of the NfL in blood derives from the Central Nervous System (CNS) (Bacioglu et al., 2016). Our study also showed that higher serum NfL levels could predict higher CSF NfL levels in patients with PD from cross-section and longitudinal studies, which was previously reported in previous studies on NfL in a variety of neurological diseases (Novakova et al., 2017). Considering that blood collection is less invasive and more maneuverable than CSF, coupled with similar diagnostic accuracy and the better predictive value of serum NfL, which we got from our study, serum NfL might act as a very early motor prognostic marker in PD.

In addition, in a mouse model of PD (transgenic A53T-α-syn mice), elevated NfL levels in CSF and serum were positively related to the number and size of neuronal α-syn inclusions (Bacioglu et al., 2016). The a-syn-formed abnormal inclusion and NfL co-localization in the brain of the mice model indicated a-syn-induced neurodegeneration could also cause NfL-related axonal injury (Sacino et al., 2014). Increased CSF NfL levels may significantly reflect α-syn abnormal accumulation (Stefanova et al., 2012). Elevated baseline CSF α-syn levels in the PD group could predict the future progression of motor symptoms. These findings confirm a direct link between α-syn deposition in disease symptoms and liquid NfL concentrations in PD pathogenesis. Abnormal aggregation of α-syn is the major pathological hallmark of PD. Therefore, NfL could mediate the dysregulation of cellular processes and function loss of central molecular mechanisms of PD by regulating α-syn activities. Our mediation analysis results also show that CSF NfL is related to the activities of α-syn. Furthermore, both NfL and tau proteins are markers of axonal degeneration, NfL seems to be a more robust marker for pathology than total tau in PD and could better represent the mortality of PD than tau (Hall et al., 2012). CSF phosphorylated tau and longitudinal changes in CSF total tau have been positively related to the worsening of motor symptoms (Hall et al., 2016). Indeed, CSF NfL levels may be related to CSF tau levels (Hall et al., 2016). These studies demonstrated that NfL could predict the motor characteristics of PD in pathophysiological and clinical features.

The strengths of this study include the use of a relatively large sample of patients with PD and HCs from a biomarker-rich database with available longitudinal clinical and multimodal neuroimaging data. In addition, we research relationships between NfL and motor indicators of PD through cross-sectional and prospective follow-up study designs. We found that the increase in NfL levels is relevant to the motor impairments of PD. In the future, NfL could be used as one of the indicators to establish a more reliable forecasting model. Besides, we found serum NfL levels strongly associated with CSF NfL levels in patients with PD and may have a better predictive value of motor impairments. Furthermore, we analyzed the difference in NfL levels in men and women with PD and may provide novel ideas for PD diagnosis and treatment. Limitations of this work first include heterogeneity in this study concerning missing values in this longitudinal cohort, particularly at subsequent follow-up time points. Secondly, the PPMI database used in this study overrepresents highly educated and Caucasian participants with access to a university hospital; it exerts a subtle effect on the results. Third, the follow-up period is three years, which is not long enough and may need further validation in more extended and extensive longitudinal studies.

Our results demonstrated that NfL levels were abnormally elevated in the individuals with PD compared with HCs. NfL, especially serum levels, could reflect disease severity in terms of both motor functions in patients with PD from the cross-sectional and longitudinal studies for the first time. Serum NfL levels also reflect CSF NfL and show a better predictive ability of motor decline in men than women with PD. The study shows that NfL, especially serum NfL, might act as a very early motor prognostic marker in PD. NfL, especially serum NfL evaluation, may be important for disease monitoring the progression of PD. But more studies are needed further to study the pathophysiological mechanisms of NfL as a predictive PD biomarker.

## Data availability statement

The original contributions presented in this study are included in the article/Supplementary material, further inquiries can be directed to the corresponding author.

## Ethics statement

The studies involving human participants were reviewed and approved by Parkinson’s Progression Markers Initiative (PPMI). The patients/participants provided their written informed consent to participate in this study.

## Author contributions

YL collected the related data, conducted the statistical analysis, and drafted the manuscript. KD contributed to the experiment design. LX and XL helped interpret the results. AX contributed in conceptualization, data curation, supervision, funding acquisition, and project administration. All authors contributed to the article and approved the submitted version.

## Footnotes

1
https://www.ppmi-info.org
